# Analysis validation has been neglected in the Age of Reproducibility

**DOI:** 10.1371/journal.pbio.3000070

**Published:** 2018-12-10

**Authors:** Kathleen E. Lotterhos, Jason H. Moore, Ann E. Stapleton

**Affiliations:** 1 Northeastern University Marine Science Center, Northeastern University, Boston, Massachusetts, United States of America; 2 Institute for Biomedical Informatics, Division of Informatics, Department of Biostatistics, Epidemiology, & Informatics, University of Pennsylvania, Philadelphia, Pennsylvania, United States of America; 3 Department of Biology and Marine Biology, University of North Carolina Wilmington, Wilmington, North Carolina, United States of America

## Abstract

Increasingly complex statistical models are being used for the analysis of biological data. Recent commentary has focused on the ability to compute the same outcome for a given dataset (reproducibility). We argue that a reproducible statistical analysis is not necessarily valid because of unique patterns of nonindependence in every biological dataset. We advocate that analyses should be evaluated with known-truth simulations that capture biological reality, a process we call “analysis validation.” We review the process of validation and suggest criteria that a validation project should meet. We find that different fields of science have historically failed to meet all criteria, and we suggest ways to implement meaningful validation in training and practice.

## Abstract 2

Just as we do controls for experiments we should all do controls for data analysis – this is easy to say but requires dedication to implement. This Essay explains the need for analysis validation and provides specific suggestions for how to get started.

## Background

The literature is awash with “re”-words: reproducibility, repeatability, replicability—even “preproducibility” [1] [2] [3] has found its way into this web of “re” (for definitions, please see Box 1). In this essay, we argue that just because a statistical analysis is reproducible or repeatable or re-whatever does not mean that it is valid. A valid statistical outcome means that the analysis has ended in a true positive or true negative result. To answer the new questions that can now be asked because the data are available, many investigators have developed new, customized data analyses and applied them to their data. However, applying a novel data analysis to an existing empirical dataset is unsatisfying because we do not know how many true positives are detected or how many false positives are generated when using empirical data. Thus, investigators need known-truth simulations to evaluate the novel statistical model they have developed. Many investigators do attempt this, but they evaluate their method under data simulated under the exact distributions and processes assumed by the method, which results in the evaluation generally favoring their method. Novel statistical methodologies need to be challenged with a variety of creative simulations that capture the spectrum of biological processes that could have created the patterns in the data. The best way to determine validity is to simulate data that capture similar patterns in the data, analyze the simulated data with various statistical approaches, and evaluate which approach yields the most accurate results. We call this process of simulation and evaluation “analysis validation,” and we assert that this is a fundamental skill for everyone, including biologists.

**Box 1. Definitions**

benchmark: something serving as a standard by which related items may be judged.

confusion matrix: table of true positives, false positives, true negatives, and false negatives that result from analysis of known-truth data. Many other metrics are available to visualize or summarize these basic tabular results, for example, the precision and recall, the sensitivity and specificity, the positive predictive value (PPV), the area under the curve (AUC), and newer measures such as the h-measure (https://www.hmeasure.net/) and plots such as the precision-recall curve (PRC).

equifinality: the idea that many processes can produce similar patterns and the same processes can produce different patterns [8].

gold standard dataset: collected, measured dataset that has the best possible information about signal and noise, i.e., best possible accuracy.

ground truth data: dataset with signal evaluated using an independent method and thus with maximum possible accuracy for a measured dataset; this term is often used in remote sensing.

known truth: term we prefer when measuring true and false positives from created datasets, whether the dataset was created using pure simulation, simulation of y given existing x values and/or distributions, or from data with signal identified from independent validation testing.

null simulation: data simulated under the null hypothesis but simulated with similar patterns of nonindependence as that observed in the real data.

overfitting: to use a statistical model that has too many parameters relative to the size of the sample, leading to a good fit with the sample data but a poor fit with new data (https://en.wiktionary.org/wiki/overfit).

repeatability: ability to make the same signals again.

replicate: to make or do or perform again, and there is a more detailed explanation at http://www.replicability.tau.ac.il/index.php/replicability-in-science/replicability-vs-reproducibility.html.

reproducibility: ability to cause to exist again, to produce again, by generation or the like, and in more detail:

“in many fields of study there are examples of scientific investigations that cannot be fully replicated because of a lack of time or resources. In such a situation, there is a need for a minimum standard that can fill the void between full replication and nothing. One candidate for this minimum standard is “reproducible research”, which requires that data sets and computer code be made available to others for verifying published results and conducting alternative analyses.” [9]

Statistical definitions and illustrations are available in Patil and colleagues [10].

simulation: the act of imitating the behavior of some situation or some process by means of something suitably analogous (especially for the purpose of study or personnel training); (computer science) the technique of representing the real world by a computer program; “a simulation should imitate the internal processes and not merely the results of the thing being simulated.”

sensitivity analysis: systematic testing of the importance of factors and factor values in generating outputs (https://www.nist.gov/sites/default/files/documents/itl/antd/philadelphiainterface052607.pdf). This concept is related to feature selection in machine learning, to effect size estimation in classical linear models, and to understanding the effects of uncertainty in parameters on the results from mathematical models.

synthetic dataset: dataset generated from a function (equation) with reproducible values for signal and noise; this is often referred to as a simulation in statistics.

true positive/false positive/true negative/false negative: the count of detected signal (true positives) and noise (true negative), with the misclassified cases as false positive and negative. The full four-way table is termed the confusion matrix (see above).

validation: finding or testing the truth of something.

The source of all definitions without references is the free dictionary, http://www.dict.org/bin/Dict.

## Why analysis validation?

Analysis validation is necessary because every biological dataset is unique in the pattern of correlation or dependency among samples. In introductory biostatistics, we teach students that individuals sampled from a population should be chosen at random (each individual chosen with equal probability) and that individuals should be independent of each other (individuals are not related to each other or interacting in some way). Unfortunately, for many biological datasets, these two properties of a good sample are violated because of logistical constraints in random sampling, because of shared ecological and evolutionary histories, and because of spatial and temporal autocorrelation. These properties of a good sample are also difficult to attain in the age of Big Data because highly dimensional datasets have unique challenges, including noise accumulation, spurious correlation, correlation between predictors and residual noise, and measurement errors [4]. Equifinality (Box 1) is ubiquitous in biological datasets. For example, statistical models that analyze patterns in DNA sequences of the human genome might make it look like those sequences have been selected for [5] [6], but models of human history that include both population growth and spatial structure can generate the observed patterns without selection [7]. Many processes can lead to the same pattern. Biologists have often used “off-the-shelf” analyses, but these rarely capture the variety of processes and sampling designs that may have generated a pattern and often have important assumptions such as multivariate normality. In some cases, even analytic approaches that are specifically designed for a type of dataset can be problematic because they make assumptions that are not met by the data. Therefore, to determine if a statistical outcome is valid, we need to better understand how accurately a statistical analysis is able to model the data.

In an attempt to confront some of these statistical issues presented by biological realism, a preponderance of new and more complex statistical methodologies are being proposed. In many cases, these methods are applied to data in which the true positives and true negatives are unknown or these methods are evaluated with data simulated following the assumptions of the method. However, it’s not always clear if the real data meet those assumptions. For this reason, the three of us, along with many of our colleagues, have started to take a more inquiry-based approach to data analysis. We mentor and teach analysis validation, which can also be stated as “test the tests.” We postulate that every experimental biology question and dataset deserves its own validation because every biological dataset and question is unique. In this essay, we provide some examples below from our personal experiences in statistical genomics, but the concepts we discuss apply to all areas of biology.

How do people get here? Scientists do a data analysis and then read about a new analysis approach, which they try…then they wonder which one is right. That leads them to designing simulations and consideration of parameter importance.—Matthew Stephens, University of Chicago

Just like we do controls for experiments, we should all do controls for data analysis. Of course, this is not easy. The nonindependence in biological systems due to shared evolutionary history, experimental design, and other limits can make thickets of constraints that have no obvious best path.

Doing an analysis validation from the beginning of a research project gives you the opportunity to justify your choices for the data you will collect and makes the statistical review process more efficient in many ways. First, documentation helps reviewers understand why you may have chosen particular settings for a statistical model that you ran on the data rather than using the default settings. Second, it helps the researcher understand what range of conclusions could be drawn from a significant result in the data. Third, it helps reviewers without statistical expertise to evaluate the results. Since there are not enough biostatisticians to review all biology grants and manuscripts, analysis validation can help streamline the review process and prevent misapplication of models in the future.

Analysis validation can be performed on many types of statistical models. These include probabilistic models, in which insights into nature can be made through parameter estimation or null hypothesis testing, as well as machine learning or algorithmic models, which aim to optimize the predictive accuracy of an algorithm rather than estimate a parameter. We advocate that the following criteria should be met for analysis validation: (i) known-truth simulations are used for evaluation of methods, (ii) the processes used to simulate data should be creative in capturing biological reality, (iii) the processes used to simulate data should not match the assumptions of one particular method, and (iv) the code for the simulations and validation should be reproducible and curated to enable future methods comparisons. Validation projects that meet these criteria will result in a better understanding of the benefits and shortcomings of different models that may be used to analyze data, and result in more robust application of models to data.

## How to validate a data analysis

### Research question and planning (Fig 1, flowchart steps 1, 2, and 3)

Before beginning a research project, explicitly formulate your research question or hypothesis and note what data are needed to answer that question. Once you identify the data necessary to answer the research question, plan for what kind of data will be collected, identify the types of statistical methods that may be appropriate, learn about their assumptions, and then assess in what ways the data may fail to meet the assumptions of those methods. All too often, investigators explicitly plan for collecting data without a detailed strategy for analyzing that data and relating the results back to the research question or hypothesis. Planning is an important part of meeting the first three of the criteria outlined in “Why analysis validation?”

**Fig 1:**
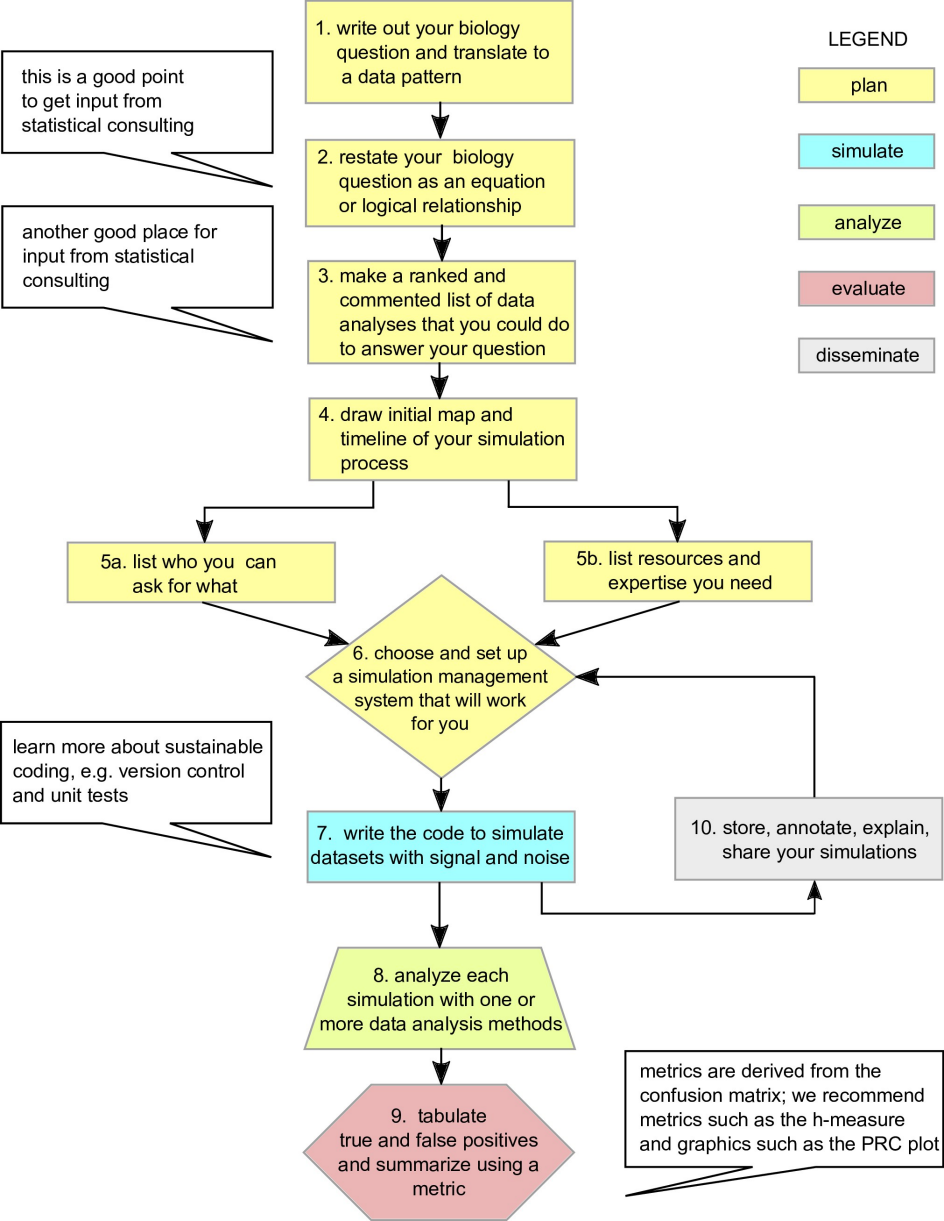
Flow chart showing some of the key steps in constructing simulations and validating data analysis methods. PRC, precision-recall curve.

### Get organized to simulate (Fig 1, flowchart steps 4, 5, and 6)

A key part of conducting a successful analysis validation study is to have a well-organized system for keeping track of simulations and results. When considering the steps in analysis validation (simulation, analysis of simulations, results from the analysis on the simulation, evaluation of the results and the calculation of performance metrics, and figures and/or visualization), a good rule of thumb is to have a folder for each of these steps in the pipeline in addition to folders for simulation and analysis scripts. Be aware that there is a random component to simulation and to some statistical approaches (for example, those based on Markov chain Monte Carlo or machine learning) and that random seeds should be specified in the pipeline so that results are reproducible. As in any analysis, proper data management and software curation standards are also required (for example, versioning, Fig 1). Since new methods are being consistently developed, it is important to structure your organization system and pipelines in such a way that new methods can be easily evaluated against previous ones, even by a person who did not contribute to the original study. In Box 2, we highlight some useful tools for performing and organizing an analysis validation study. Being organized is an important part of meeting the final criterion outlined in “Why analysis validation?”

**Box 2. Validation management tools**

For each example below, we highlight the special feature that was used to focus the design.

- DSC, Dynamic Statistical Comparisons: https://stephenslab.github.io/dsc-wiki/, https://github.com/stephens999/dscr/blob/master/intro.md. This tool organizes inputs and outputs for simulation and analysis and leverages powerful Script-of-Scripts pipelines, with a focus on enabling the user to add new functionality easily. This program priority is to be “extensible”: to easily allow additional methods and simulations.
- The simulator R package (https://arxiv.org/abs/1607.00021, http://faculty.bscb.cornell.edu/~bien/simulator.html) can be used to track simulation processes and handle mechanics such as random numbers and plotting, with a modular specification of what the user would need to consider for simulations, analyses, and metrics. This program priority is to be “manageable”: to allow users to easily keep track of the different processes and stages in their simulations.
- CyVerse Validate (http://validate-10.readthedocs.io/en/latest/) uses public computational resources (for democratic access to resources) and a generalizable computation management tool (Agave, https://tacc-cloud.readthedocs.io/projects/agave/en/latest/ for genome-wide association studies (GWAS) tool comparisons. This project priority is to be “scalable”: to allow researchers to compare analyses and manage running of simulations using public compute resources that accommodate increased computational demand.

From the Machine Learning community, we highlight repository examples that illustrate how simulations can be shared:

- The Penn Machine Learning Benchmark (PMLB) at https://github.com/EpistasisLab/penn-ml-benchmarks. This effort leverages github functionality to track use and curate the code and data.
- Image analysis (for example, ImageNet, image-net.org) repositories are used for storage of gold standard datasets and use links and publications to track usage and results.

There are many other tools and repositories available. We encourage readers to post additional suggestions in the comments section associated with this essay, noting use cases and target user and contributor communities.

### Plan simulations, find necessary expertise and resources (Fig 1, flowchart steps 5, 7, and 8)

Once you have planned how to organize your validation project, ask yourself the following:

- If I simulate data under the null (or some other) hypothesis and analyze it the same way as the real data, what results do I get?
- If I make changes to the simulations, how does the output change? What increases false positives and false negatives?

With complex situations, the null simulation could be hard to decide on, so your experience with the system and your specific question will assist in prioritizing the number and range of simulations you plan to do (Fig 1). Don’t plan to simulate data that follow the exact model of the statistical method being evaluated; instead, consider the types of nonindependence in the real data and consider different process that could produce the same pattern. Designing simulations will be important for meeting the second and third criteria outlined in “Why analysis validation?” This range helps you determine what the key factors/parameters are in your system and may prompt you to answer new questions in your next set of experiments. Simulations let you explore your system and help you optimize resources for biological verification. A simulation project should normally include both the equations and theory, the code and functions you used to create the data, and the output files that result from running the code (though in some cases, it is not optimal to store large output files, and options for reproducible rerunning can be provided instead).

Nuances in data analysis can affect the outcomes of studies; important details include data filtering, imputation of missing data values, and model selection approaches. These should be included in simulations in ways that reflect the experimental design and data collection. Since it is time consuming and often impossible to reproduce the results without the analysis scripts, curation of simulations and analysis methods in reproducible pipelines is an important component of a validation project (Box 2, and necessary to meet the fourth criterion outlined in “Why analysis validation?”). Doing the simulations also ensures you thoroughly understand how to do the data analysis, which will save you time later when you have your experimental results.

### Compare the results and calculate performance metrics for different statistical models (Fig 1, flowchart step 9)

After the simulations have been analyzed with different statistical models, the known truth of the simulations can be used to compare the performance of the different models. Performance metrics come in two flavors: those based on a confusion matrix for a single cutoff (for example, a cutoff that gives 1% false positives or a cutoff P-value), and those based on comparison of all thresholds, such as the area under the curve of the precision-recall graph (AUC-PR) [11]. Performance metrics based on the confusion matrix for a single cutoff can sometimes be misleading because a method that performs better than another at one cutoff may perform worse at a different cutoff. The AUC is generally preferred because it integrates over all possible cutoffs for the data.

### Simulation management and curation (Fig 1, flowchart steps 6 and 10)

Beyond managing your own work, there is real synergy in working with shared, community simulation repositories, as we have seen in the fields of machine learning and image analysis. The evolution community has called for a bank of simulations that can be used to validate or verify methods, https://www.nescent.org/cal/calendar_detail.php-id=1105.html, as has the National Institutes of Health (NIH) workshop on genetic simulation [12]. Consider how your simulations will be curated and shared (criterion four in “Why analysis validation?”)—your work does have value for others!

## Evolution of analysis validation across fields

Across different fields, the criteria for analysis validation varies. Here, we review whether current practices typically meet the four criteria outlined in “Why analysis validation?” While we recognize that analysis validation is a process in which models and simulations necessarily start simply and become more and more complex as knowledge accumulates, below, we show that despite knowledge increasing rapidly, there is still a lag in the rate this knowledge is incorporated into testing methods.

Computer scientists a have a long-standing culture of using public datasets for the development and evaluation of machine learning methods. The computer science goal is to answer questions about algorithm performance relative to prior algorithms, so computer scientists are naturally more interested in algorithms and easy access to benchmark data. A widely used source of public data is the UCI Machine Learning Repository from the University of California Irvine that has been available since 1987 (http://archive.ics.uci.edu/ml). This repository currently has more than 400 datasets from a wide range of different disciplines, including nearly 100 from the biological and biomedical sciences. Other sets of benchmark data have been assembled, such as the Penn Machine Learning Benchmark (PMLB) resource from the University of Pennsylvania that includes both public and simulated data [13]. While these resources are generally very useful for methods comparisons, they aren’t without problems. For example, it is difficult to benchmark and compare algorithms with real data since the truth is not known. Further, it is often not known or understood how the data were generated and what the quality control issues are. It is also common to evaluate algorithms on a subset of the datasets, which raises questions about whether the data used were selected to maximize performance of the method being developed. These issues and others were raised in a recent community survey of best practices for those working on a type of machine learning called genetic programming [14]. This paper went as far as to recommend blacklisting some benchmarks because they were too easy, out of date, or not appropriate for the methods being developed. As the number of statistical models increase, the number of benchmarks does not, especially for experimentally validated datasets, which are difficult and expensive to generate—which increases the risk of overfitting because more and more models are developed, but only the ones that show a significant improvement are published. In other words, testing an increasing number of methods on a small group of datasets until you discover a better model is analogous to running a large number of statistical models on one dataset until you find one that is significant. Thus, common current practice in computer science fails to meet our first three criteria for analysis validation because the truth is unknown for the benchmark datasets.

In contrast, statisticians normally create and use simulations, as well as using public datasets, for new method development. However, statisticians do not have sharing tradition, a widely used common repository for simulations, or common metadata standards for simulations. There are some efforts to develop repositories, such as the workshop and repository for genome-wide analysis simulations described by Peng and colleagues [15] [12], but this is not widespread. Typically, statistics instructors create simulations to use in class, but they are not often shared or scalable, and learning simulation construction is not typically part of the undergraduate statistics curriculum. In 2015–2016, the need for simulation management was addressed by statisticians, and since then, there have been some development efforts for management systems (Box 2). The field of statistics thus provides a great example of using simulations to evaluate methods (our first criterion) but does not meet our other criteria because simulations often follow assumptions of the methods, and publications do not always include the code to rerun the simulations. Only biostatisticians would be focused on our second criteria (creative simulations); we advocate for more biostatistician–biologist partnerships in development of simulation systems that are statistically sound and biologically relevant.

Ecology and evolutionary biology both have a long tradition of simulations for analysis method development, but like statisticians, these scientists have not historically shared simulations and code. For example, method comparisons in landscape ecology produced a paper with over 4,000 citations since 2006 [16] and the establishment of a preferred method with over 5,500 citations [17]. Typically, however, these fields of biology have grown through the cyclical process of proposing a statistical model, recognizing that the model is insufficient, proposing a new model and validating the model with simulated data, recognizing that the simulated data were overly simplistic, proposing more realistic simulations and models, and so on. A good example of how this cyclical process has played out in evolutionary biology is for a statistical test called an F_ST_ outlier test, which seeks to identify which loci in a genome are under selection and which are neutral. In 1973, Lewontin and Krakeur [18] were the first to propose that unusually large or “outlier” values of F_ST_ indicate that the locus may be affected by selection. Difficulties with the method were immediately recognized, however, because the variation in F_ST_ depends on sample sizes and the degree of independence of the evolutionary histories of sampled populations [19] [20] [21]. The next major improvement on the F_ST_ outlier test was to account for differences in sample size [22], which became a widely used method (cited >1,500 times) despite the fact that the model was evaluated on relatively simple simulations and the model still did not account for varying degrees of nonindependence among sampled populations. Analysis validation on this method with more realistic simulated data was able to clearly illustrate how shared evolutionary history (a source of nonindependence among samples) caused the method to have many false positives [23]. Recently, many flavors of F_ST_ outlier tests have been evaluated with more realistic simulations, and they have much lower false positive rates because they control for nonindependence among populations in the calculation of significance of test statistics [24] [25] [26] [27]. We note that the standards for our first three criteria have improved over time in the fields of ecology and evolutionary biology, but these fields still fail to use shared repositories or benchmarks for evaluation (the fourth criterion).

Bioinformaticians and statistical genomicists typically share simulation results and pseudocode rapidly (for example, biostars https://www.biostars.org/, SEQanswers http://seqanswers.com/) but do not routinely use shared repositories or shared design patterns for simulations. As in ecology and evolutionary biology, comparisons of methods are often done by specialists and published as static results. Bioinformatics and statistical genomics researchers have been slow to evaluate methods against biologically realistic simulations, which is in part due to the computational challenge of simulating genomes and the process of simulating next-generation sequencing because simulation tools have only recently become available [28, 29, and 30]. Because of the latency in generating known-truth simulations in these fields, there is still much to be learned about the consequences of violating the assumptions of statistical models for genomic data analysis. For example, a common goal of many genomics studies is to identify the genetic markers that increase risk for common human diseases, which are often identified with statistical tests called genome-wide association studies (GWASs). Modern human GWASs were developed in the mid-2000s with the availability of chips for genotyping [31] [32] and quickly converged on a standard protocol for data processing and analysis that helped facilitate the reduction of false positives and an increase in the replication of results across multiple studies. While the human GWAS approach has had success identifying numerous genetic risk factors [33], there are very few discoveries that have led to new prevention and treatment strategies. It is generally recognized that many real genetic risk factors may have been missed because the rigid analytical approach makes many important assumptions that might not be valid. For example, typical human GWASs assume that each single-nucleotide polymorphism (SNP) has an additive effect on risk that is independent of genomic and ecological context (for example, a univariate linear model), an assumption that is increasingly being questioned for complex disease traits [34]. There is also an extensive list of numerous other statistical assumptions that may not make sense given the enormous complexity of common human diseases. Violation of any of these assumptions could invalidate the data analysis method, at which point an independent experimental biological validation (for example, gene editing) becomes pointless. Our experience with GWASs has thus taught us that careful listing and scrutiny of all the assumptions of the analysis, and how the data may violate them, is an important component of designing simulations for analysis validation. Thus, while bioinformatics and statistical genomics have a history of creating simulations for methods evaluation (the first criterion), these simulations are in many cases overly simplistic and do not often capture biological reality and are not typically shared in common repositories, so they fail to fulfill the remaining criteria.

Across many fields, contests have historically played and continue to play an important role in analysis method development and in teaching and learning about validation. Open competitions for data analysis have a long history and have been key in the development of protein structure prediction methods (http://predictioncenter.org/index.cgi), in early microarray data analysis (http://camda.duke.edu/), and in association and/or quantitative trait locus (QTL) analysis (https://www.gaworkshop.org/, http://qtl-mas-2012.kassiopeagroup.com/en/index.php). Some examples of competition-based innovations that have moved the whole field forward include the dialogue for reverse engineering assessments and methods (DREAM) challenge for pathway inference and networks in biology and medicine (http://dreamchallenges.org/project/dream-5-network-inference-challenge/) and the protein structure effort WeFold [35] (https://wefold.nersc.gov/wordpress/). We highlight DREAM and WeFold because they have contributed an emphasis on collaboration—with tools developed to support team interaction and with incentives to improve teamwork during challenges. The open release of simulated data in the QTL-marker–assisted selection (QTL-MAS) project and for experimentally determined structures in critical assessment of techniques for protein structure prediction (CASP) has also benefited the community of researchers in these areas. Challenges are also used as explicit teaching and learning tools. For example, the software and statistical methods for population genetics (SSMPG) workshop uses a data challenge as a way to learn how to analyze a genome for the regions involved in local adaptation (https://github.com/bcm-uga/SSMPG2017). The students are presented with a simulated dataset (with known true positives and true negatives) and receive several tutorials on statistical methods that may be used to analyze the data. Students are then given free time to analyze the simulated data as they see fit and submit their results to a website that returns a score based on the number of true and false discoveries in their submission. The website posts the scores on a leaderboard, and students can work to improve their score through reanalysis of the data. Through this process, they learn about both the strengths and weaknesses of the different methods, the best practices for analyzing the data, and how analysis validation works. Academic and commercial contests have moved toward increased code and simulation sharing in recent years and could provide effective incentives and training relevant to all four of our criteria if the contest designers prioritize those aspects.

## Shortcomings of other approaches for evaluation

Often, biologists are reluctant to undertake a simulation project because they don’t have the expertise or they think it unfeasible, and so they evaluate and compare methods on real data instead. This can be accomplished through a sensitivity analysis and/or cross-validation. Note that neither of these approaches can give insight into whether a particular outcome gives a true positive or false positive result. Thus, although these approaches have some benefits and caveats discussed below, both of these approaches fail to meet the first and most important criterion for analysis validation (using simulations).

A sensitivity analysis determines how robust the results are to different decisions made while analyzing the data. In the field of genomics, for example, it may be how sensitive results are to the way missing genotypes are interpolated for analysis, while in the field of landscape ecology, it may be how sensitive results are to the way data are interpolated across spatial locations. Sensitivity analysis has the benefit of identifying particular choices or parameters that may influence outcomes.

Cross-validation is a procedure that partitions the data into a training set that is used to fit model parameters and a test set that is used to measure the prediction errors. However, cross-validation is just measuring error of predictions—therefore, it measures precision and not accuracy. If your data violate the assumptions of the model and you get the wrong result, you can still have a precise cross-validation that is not accurate (i.e., all darts hit the same place on the dartboard, but they are far from the bullseye). Because in biology, many different processes can create the same pattern, a model can perform well in a cross-validation but still model a different process from the one that created the data. Analysis validation can help resolve disputes on how the output of statistical models should be interpreted, even when they perform well in cross-validation (for example, [36]).

Cross-validation with real data is also problematic for comparing the performance of statistical and machine learning methods because the true signal in the data is unknown. One method may appear to perform better than another on a real dataset simply because it is doing a better job of modeling systematic noise or error rather than true signal. This can yield misleading claims of method performance, which in turn can negatively influence studies that adopt these methods. We recommend that comparison of methods be first performed using simulated data in which the truth is known. The challenge here is to simulate realistic patterns that appropriately challenge the methods. One clear advantage of simulation for method comparison is that it is relatively easy to simulate data with differing sample sizes, numbers of variables or features, varying amounts of noise, varying effect sizes, varying pattern complexity, etc. The ability to vary simulation parameters makes it much easier to determine the strengths and weaknesses of different methods. We recommend that real data be used with cross-validation or other similar methods only after a comprehensive simulation study has been completed. Any comparisons with real data should be followed by an in-depth interpretation of the results to compare the biological plausibility of the models that are generated in addition to standard estimates of error that are provided by cross-validation.

## A path toward validation for all

We postulate that simulation and validation is likely to be a key skill to teach early, the same way we teach lab notebooks, protocols, and experimental design in the natural sciences (for example, http://www.lifescied.org/content/13/2/265 and https://www.lifescied.org/doi/abs/10.1187/cbe.13-11-0218). Colon-Berlingeri and Burrowes, for example, describe the use of simulated data in biology curricula [37]. Many biology faculty have used simulation modules to teach experimental design and data analysis to undergraduates, historically using EcoBeaker and Virtual Flylab (Desharnais and Bell, http://www.imej.wfu.edu/articles/1999/2/01/printver.asp) and now Simbio (http://simbio.com/promos/experimental-design). It would be very helpful to have additional materials available for other audiences along with teaching materials that are specific to analysis validation. It is abundantly clear that open source is the sustainable path forward in making analysis and simulation tools available to everyone.

In addition to supporting use of open source software, graduate programs should already be requiring trackable electronic notebooks for all students and policies for review of these; additional development of these policies would be a great opportunity for collaboration with research compliance offices. A requirement like this is already in place for the graduate program in Genomics and Computational Biology at the University of Pennsylvania, where students have their electronic notebooks reviewed by the graduate committee at each annual meeting. Notebooks are reviewed for completeness with respect to manuscripts and published papers in which the ability to reproduce the work of the student is an important consideration. Incomplete notebooks are reported to the chair of the graduate program and the adviser. There are several benefits to this kind of policy beyond ensuring reproducibility of the results. First, it can lead to an improved mentoring relationship with the student. Second, it encourages the student to more carefully document their work beyond what might be required to reproduce a result. Third, it fosters developmental rather than punitive tracking of the student’s progress. Training in data curation (one source for more detail on data about data is http://www.datacarpentry.org/organization-genomics/01-tidiness/) is also likely to be valuable for all, and we advocate for more dialog with information schools and librarians in theory and practice for development of scalable curation teaching methods and tools as a complement to specific training in simulation for validations. Doing simulations early makes data analysis and peer review more efficient, which benefits students as they progress toward their degrees and has the potential to improve their career trajectories.

A longer-term advantage of simulation is the potential to characterize repositories of simulations using mathematical similarity, as well as using text-based or manual ontologies; this would allow building on existing efforts such as ontology development (Coehlo and colleagues 2012, https://bibliotecadigital.fgv.br/dspace/bitstream/handle/10438/15033/Towards_an_Ontology_for_Mathematical_Modeling_with_Application_to_Epidemiology.pdf), automated analysis such as OntoMathPro (http://ontomathpro.org/ontology/), and exploiting equation libraries such as the Digital Library of Mathematical Functions (https://dlmf.nist.gov/).

The rise in simulations is expected to drive new computer science research and applications (https://upcommons.upc.edu/handle/2117/96661). Novel methods for incentivizing collaboration and simulation and new ways to support long-term curation, taxonomic grouping in repositories, and search are needed. Every field calls analysis validation something slightly different (Box 1), which impedes search serendipity and thus dissemination. One straightforward early step would be workshops and hackathons for simulation-management tool developers that are coordinated with gateway cyberinfrastructure developers (https://sciencegateways.org/), so as to better connect scientists with scalable open access computational resources, and design and implementation input from curation system experts such as ontologists and librarians.

We suggest starting with a focus on designing simulation repositories that update gracefully, that leverage shared libraries for simulation features such as missingness, noise, and equation taxonomy, and that can be run with original and novel analysis code on public computational resources. Lessons learned from existing image repositories will be valuable in designing new simulation repositories. We also recommend that both sustainability and adaptability of any curation and repository effort be a notable component of funding. We advocate for a living, widely used repository system that scales well and leverages volunteer contributions in an exemplary way. Ideally, fields would agree on benchmark simulations that would be used to measure performance on all developed methods. In our experience, the fields of ecology and quantitative genetics have excellent individual examples of scientists who combine modeling and experimentation. We encourage deeper integration of these complementary skills for all biologists at all levels.

## Abbreviations

AUC: area under the curve
CASP: critical assessment of techniques for protein structure prediction
DREAM: dialogue for reverse engineering assessments and methods
DSC: Dynamic Statistical Comparisons
GWAS: genome-wide association study
NIH: National Institutes of Health
PMLB: Penn Machine Learning Benchmark
PPV: positive predictive value
PR: precision recall
PRC: precision-recall curve
QTL: quantitative trait locus
QTL-MAS: QTL-marker–assisted selection
SNP: single-nucleotide polymorphism
SSMPG: software and statistical methods for population genetics
UCI: University of California Irvine

## References

[1] Leek JT, Jager LR. Is Most Published Research Really False? Annual Review of Statistics and Its Application. Annual Reviews; 2017;4: 109–122. 10.1146/annurev-statistics-060116-054104

[2] Baker M. 1,500 scientists lift the lid on reproducibility. Nature. 2016;533: 452–4. 10.1038/533452a 27225100

[3] Stark PB. Before reproducibility must come preproducibility. Nature. Springer Nature; 2018;557: 613–613. 10.1038/d41586-018-05256-0 29795524

[4] Fan J, Han F, Liu H. Challenges of Big Data analysis. National Science Review. Oxford University Press (OUP); 2014;1: 293–314. 10.1093/nsr/nwt032 25419469 PMC4236847

[5] Mekel-Bobrov N. Ongoing Adaptive Evolution of ASPM a Brain Size Determinant in Homo sapiens. Science. American Association for the Advancement of Science (AAAS); 2005;309: 1720–1722. 10.1126/science.1116815 16151010

[6] Evans PD. Microcephalin a Gene Regulating Brain Size, Continues to Evolve Adaptively in Humans. Science. American Association for the Advancement of Science (AAAS); 2005;309: 1717–1720. 10.1126/science.1113722 16151009

[7] Currat M. Comment on Ongoing Adaptive Evolution of ASPM a Brain Size Determinant in Homo sapiens and Microcephalin a Gene Regulating Brain Size, Continues to Evolve Adaptively in Humans. Science. American Association for the Advancement of Science (AAAS); 2006;313: 172a–172a. 10.1126/science.1122712 16840683

[8] Bertalanffy Lvon. General System Theory: Foundations, Development, Applications. 1969.

[9] Peng RD. Reproducible research and Biostatistics. Biostatistics. Oxford University Press (OUP); 2009;10: 405–408. 10.1093/biostatistics/kxp014 19535325

[10] Patil P, Peng RD, Leek J. A statistical definition for reproducibility and replicability. BioRxiv 066803 [Preprint]. 2016 [cited 2016 July 29]. Available from: https://www.biorxiv.org/content/early/2016/07/29/066803

[11] Davis J, Goadrich M. The relationship between Precision-Recall and ROC curves. Proceedings of the 23rd international conference on Machine learning—ICML 06; Pittsburgh, PA, USA. New York: ACM Press; 2006. doi:10.1145/1143844.1143874

[12] Chen H-S, Hutter CM, Mechanic LE, Amos CI, Bafna V, Hauser ER, et al Genetic Simulation Tools for Post-Genome Wide Association Studies of Complex Diseases. Genetic Epidemiology. Wiley; 2014;39: 11–19. 10.1002/gepi.21870 25371374 PMC4270837

[13] Olson RS, La CW, Orzechowski P, Urbanowicz RJ, Moore JH. PMLB: a large benchmark suite for machine learning evaluation and comparison. BioData Min. 2017;10: 36 10.1186/s13040-017-0154-4 29238404 PMC5725843

[14] White DR, McDermott J, Castelli M, Manzoni L, Goldman BW, Kronberger G, et al Better GP benchmarks: community survey results and proposals. Genetic Programming and Evolvable Machines. Springer Nature; 2012;14: 3–29. 10.1007/s10710-012-9177-2

[15] Peng B, Chen H-S, Mechanic LE, Racine B, Clarke J, Gillanders E, et al Genetic Data Simulators and their Applications: An Overview. Genetic Epidemiology. Wiley; 2014;39: 2–10. 10.1002/gepi.21876 25504286 PMC4804465

[16] Elith J, Graham CH, Anderson RP, Dudík M, Ferrier S, Guisan A, et al Novel methods improve prediction of species’ distributions from occurrence data. Ecography. Wiley; 2006;29: 129–151. 10.1111/j.2006.0906–7590.04596.x

[17] Phillips SJ, Anderson RP, Schapire RE. Maximum entropy modeling of species geographic distributions. Ecological Modelling. Elsevier BV; 2006;190: 231–259. 10.1016/j.ecolmodel.2005.03.026

[18] Lewontin RC, Krakauer J. Distribution of gene frequency as a test of the theory of the selective neutrality of polymorphisms. Genetics. 1973;74: 175–95. 4711903 10.1093/genetics/74.1.175 PMC1212935

[19] Lewontin RC, Krakauer J. Letters to the editors: Testing the heterogeneity of F values. Genetics. 1975;80: 397–8. 1132692 10.1093/genetics/80.2.397 PMC1213337

[20] Robertson A. Letters to the editors: Remarks on the Lewontin-Krakauer test. Genetics. 1975;80: 396 1132691 10.1093/genetics/80.2.396 PMC1213336

[21] Nei M, Maruyama T. Letters to the editors: Lewontin-Krakauer test for neutral genes. Genetics. 1975;80: 395 1132690 10.1093/genetics/80.2.395 PMC1213335

[22] Beaumont MA, Nichols RA. Evaluating Loci for Use in the Genetic Analysis of Population Structure. Proceedings of the Royal Society B: Biological Sciences. The Royal Society; 1996;263: 1619–1626. 10.1098/rspb.1996.0237

[23] Lotterhos KE, Whitlock MC. Evaluation of demographic history and neutral parameterization on the performance of FSToutlier tests. Molecular Ecology. Wiley; 2014;23: 2178–2192. 10.1111/mec.12725 24655127 PMC4228763

[24] Whitlock MC, Lotterhos KE. Reliable Detection of Loci Responsible for Local Adaptation: Inference of a Null Model through Trimming the Distribution of FST. The American Naturalist. University of Chicago Press; 2015;186: S24–S36. 10.1086/682949 26656214

[25] Luu K, Bazin E, Blum MGB. pcadapt: an R package to perform genome scans for selection based on principal component analysis. Mol Ecol Resour. 2017;17(1):67–77. Epub 2016 Sep 7. 10.1111/1755-0998.12592 27601374

[26] Gautier M. Genome-Wide Scan for Adaptive Divergence and Association with Population-Specific Covariates. Genetics. 2015 12;201(4):1555–79. Epub 2015 Oct 19. 10.1534/genetics.115.181453 26482796 PMC4676524

[27] Fariello MI, Boitard S, Naya H, SanCristobal M, Servin B. Detecting Signatures of Selection Through Haplotype Differentiation Among Hierarchically Structured Populations. Genetics. Genetics Society of America; 2013;193: 929–941. 10.1534/genetics.112.147231 23307896 PMC3584007

[28] Messer PW. SLiM: Simulating Evolution with Selection and Linkage. Genetics. Genetics Society of America; 2013;194: 1037–1039. 10.1534/genetics.113.152181 23709637 PMC3730910

[29] Peng B, Kimmel M. simuPOP: a forward-time population genetics simulation environment. Bioinformatics. Oxford University Press (OUP); 2005;21: 3686–3687. 10.1093/bioinformatics/bti584 16020469

[30] Escalona M, Rocha S, Posada D. A comparison of tools for the simulation of genomic next-generation sequencing data. Nature Reviews Genetics. Springer Nature; 2016;17: 459–469. 10.1038/nrg.2016.57 27320129 PMC5224698

[31] Hirschhorn JN, Daly MJ. Genome-wide association studies for common diseases and complex traits. Nat Rev Genet. 2005;6: 95–108. 10.1038/nrg1521 15716906

[32] Wang WYS, Barratt BJ, Clayton DG, Todd JA. Genome-wide association studies: theoretical and practical concerns. Nature Reviews Genetics. Springer Nature; 2005;6: 109–118. 10.1038/nrg1522 15716907

[33] Visscher PM, Wray NR, Zhang Q, Sklar P, McCarthy MI, Brown MA, et al 10 Years of GWAS Discovery: Biology, Function, and Translation. Am J Hum Genet. 2017;101: 5–22. 10.1016/j.ajhg.2017.06.005 28686856 PMC5501872

[34] Sanjak J, Long AD, Thornton KR. A model of compound heterozygous loss-of-function alleles is broadly consistent with observations from complex-disease GWAS datasets. PLoS Genet. 2017 1 19;13(1):e1006573 10.1371/journal.pgen.1006573 28103232 PMC5289629

[35] Keaser C, McGuffin LJ, Wallner B, Chopra G, Adhikari B, Bhattacharya D, et al An analysis and evaluation of the WeFold collaborative for protein structure prediction and its pipelines in CASP11 and CASP12. Scientific Reports. 2018;8:9939 10.1038/s41598-018-26812-8 Available from: https://www.nature.com/articles/s41598-018-26812-8. 29967418 PMC6028396

[36] Fitzpatrick MC, Keller SR, Lotterhos KE. Comment on “Genomic signals of selection predict climate-driven population declines in a migratory bird”. Science. http://science.sciencemag.org/content/361/6401/eaat7279/tab-article-info; 2018;361 Retrieved: http://science.sciencemag.org/content/361/6401/eaat7279 10.1126/science.aat7956 30072513

[37] Colon-Berlingeri M, Burrowes PA. Teaching Biology through Statistics: Application of Statistical Methods in Genetics and Zoology Courses Jungck JR, editor. CBELife Sciences Education. American Society for Cell Biology (ASCB); 2011;10: 259–267. 10.1187/cbe.10-11-0137 PMC3164565 21885822

